# Dietary Polyphenols and Cardiovascular Health: From Bioavailability to Clinical Relevance

**DOI:** 10.3390/molecules31193549

**Published:** 2026-10-05

**Authors:** Maja Krstić Ristivojević, Dušan Ružičić, Monica Trif, Sofia Papadaki, Petar Ristivojević, Ilija Cvijetić

**Affiliations:** 1 Department of Biochemistry, Faculty of Chemistry, University of Belgrade, Studentski trg 12–16, 11000 Belgrade, Serbia; krstic_maja@chem.bg.ac.rs; 2 Department of Invasive Cardiology, Health Center Valjevo, 14000 Valjevo, Serbia; ducaruzicic@yahoo.com; 3 Faculty of Health Sciences, Singidunum University, 14000 Valjevo, Serbia; 4 Centre for Innovative Process Engineering (CENTIV) GmbH, 28857 Syke, Germany; monica_trif@hotmail.com; 5 Department of Natural Resources and Agricultural Engineering, School of Environment and Agricultural Engineering, Agricultural University of Athens, 75 Iera Odos, 118 55 Athens, Greece; papadaki@aua.gr; 6 PRINSUS P.C., Iron Polytechniou 9, Zografou Campus, 157 80 Athens, Greece; 7 Department of Analytical Chemistry, Faculty of Chemistry, University of Belgrade, Studentski trg 12–16, 11000 Belgrade, Serbia; ristivojevic@chem.bg.ac.rs

**Keywords:** dietary polyphenols, cardiovascular disease, bioavailability, gut microbiota, phenolic metabolites, endothelial function, clinical translation, precision nutrition

## Abstract

Dietary polyphenols, abundant in fruits, vegetables, tea, coffee, cocoa, and wine, have been widely investigated for their potential relevance to cardiovascular health. Their proposed protective effects extend beyond classical antioxidant activity, encompassing regulation of redox-sensitive signaling, attenuation of inflammation, improvement in endothelial function, modulation of lipid metabolism, and mitochondrial protection. Yet, their biological impact is strongly dependent on bioavailability and metabolic fate, as most compounds undergo extensive conjugation and gut microbiota-dependent transformation. Conjugated metabolites and microbial derivatives increasingly appear as key mediators of systemic vascular responses. Epidemiological studies associate polyphenol-rich dietary patterns with reduced cardiovascular risk, while clinical trials report modest and variable effects on intermediate outcomes such as vascular function, blood pressure, and plasma lipids. However, heterogeneity in study design, dosing, and compound source contributes to inconsistent outcomes and limits translation into practice. This review integrates current knowledge on classification, dietary sources, metabolism, and molecular mechanisms of polyphenols, emphasizing the interplay between host and microbiota in shaping cardioprotective responses. By bridging mechanistic insights with clinical observations, we highlight both the therapeutic promise and the translational challenges of polyphenols. Standardized intervention protocols, physiologically relevant dosing strategies, and precision nutrition approaches are essential to fully realize their potential in cardiovascular prevention.

## 1. Introduction

Cardiovascular diseases (CVD) remain the leading cause of morbidity and mortality worldwide. According to the World Health Organization, approximately 17.9 million deaths are attributed to CVDs annually, accounting for 32% of all global deaths, with nearly 85% resulting from myocardial infarction and stroke. The burden is unevenly distributed across regions and populations. In 2019, Central Europe, Eastern Europe, and Central Asia showed the highest age-standardized CVD mortality rates, reaching 524.1 and 345.7 deaths per 100,000 men and women, respectively [1,2].

Hypertension remains one of the most important risk factors for CVD, affecting approximately 1.28 billion adults worldwide, while nearly half of affected individuals remain undiagnosed. Although pharmacological treatment has substantially improved cardiovascular outcomes, the global burden of CVD remains high and continues to affect mostly low- and middle-income countries. This highlights the need for preventive strategies that extend beyond conventional therapy. Because many cardiovascular risk factors are closely linked to lifestyle, particularly dietary habits, preventive nutrition has become an important area of cardiovascular research. Within this context, food-derived bioactive compounds, including dietary polyphenols, have attracted increasing attention as potential contributors to cardiovascular prevention [3,4].

Polyphenols are abundant in commonly consumed foods, including fruits, vegetables, whole grains, tea, coffee, cocoa, and red wine. Early interest in dietary polyphenols was largely driven by epidemiological observations such as the “French Paradox,” which described a relatively low incidence of coronary heart disease in populations consuming diets rich in saturated fats but also regularly consuming red wine [3]. Although this observation was initially linked to the antioxidant properties of grape-derived polyphenols, particularly their ability to inhibit low-density lipoprotein (LDL) oxidation, later research has broadened this view. Experimental studies have shown that polyphenols can modulate cell signaling, gene expression, enzyme activity, receptor-mediated responses, and host–microbiota interactions [4].

Experimental studies suggest that polyphenols can influence endothelial function, redox-sensitive and inflammatory signaling, mitochondrial homeostasis, lipid metabolism, platelet activity, and host–microbiota interactions [4,5,6,7,8,9]. However, many mechanistic studies use unconjugated parent compounds at concentrations exceeding those achieved in humans. Following ingestion, polyphenols undergo extensive host and microbial metabolism and circulate mainly as conjugated or microbiota-derived metabolites. This difference between experimental and human exposure, together with substantial interindividual metabolic variability, limits the direct translation of mechanistic findings into clinical cardiovascular benefit.

Findings from experimental studies are increasingly supported by clinical and epidemiological evidence. Polyphenol consumption has been associated with lower cardiovascular risk, modulation of lipid metabolism, improved vascular and endothelial function, and reduced platelet activation [10,11]. Meta-analyses further suggest beneficial effects on blood pressure regulation, nitric oxide bioavailability, and atherosclerosis-related processes [12].

Nevertheless, important limitations remain. Many experimental studies employ supraphysiological concentrations, while human intervention studies often vary substantially in design, dose, duration, and polyphenol source, contributing to inconsistent clinical outcomes and limiting definitive therapeutic recommendations [6,11].

To contextualize the scope of the present review, Table 1 summarizes the main scope and key aspects addressed by previous reviews and the present review, including polyphenol bioavailability and metabolism, mechanisms of action, and human/clinical evidence. The table provides a descriptive comparison of selected representative reviews and is intended to illustrate differences in scope and emphasis rather than to rank their quality or completeness.

Therefore, this review aims to: (i) classify polyphenols according to chemical properties, biological activities, and dietary sources; (ii) critically discuss the role of bioavailability, metabolism, and gut microbiota in shaping the biological activity and cardiovascular efficacy of dietary polyphenols; (iii) integrate recent evidence on the interactions between polyphenols, intestinal microbiota, and host metabolic pathways as key determinants of interindividual variability in cardioprotective responses; (iv) summarize the molecular mechanisms underlying cardioprotective effects beyond antioxidant activity, with particular emphasis on mitochondrial protection, endothelial signaling, inflammation, epigenetic regulation, and redox-sensitive pathways; and (v) evaluate translational evidence from human studies, highlighting current limitations, inconsistencies, and future directions for precision nutrition approaches in cardiovascular prevention.

Unlike previous reviews that primarily focused on isolated mechanisms or specific subclasses of polyphenols, this review provides an integrated perspective linking bioavailability, microbiota-dependent metabolism, molecular cardioprotective mechanisms, and translational relevance. Furthermore, special attention is given to the gap between promising experimental findings and inconsistent clinical outcomes, emphasizing the need for standardized intervention protocols, physiologically relevant dosing strategies, and personalized nutrition frameworks for cardiovascular health optimization.

### Literature Search and Selection

This critical narrative review was informed by structured searches of PubMed, CAS SciFinder-n, and Google Scholar. The searches covered literature available through 1 August 2026, with particular emphasis on original research published from January 2016 onward.

The search strategy used terms related to the main topics of the review, including “dietary polyphenols,” “cardiovascular disease,” “cardiovascular protection,” “bioavailability,” “bioaccessibility,” “intestinal absorption,” “Phase II metabolism,” “gut microbiota,” “microbial metabolism,” “phenolic metabolites,” “urolithins,” “endothelial function,” “oxidative stress,” “inflammation,” “blood pressure,” “lipid metabolism,” “polyphenol supplementation,” and “randomized controlled trials.” These terms were searched individually and in relevant combinations. The reference lists of key publications were also examined to identify additional studies.

Elicit was used as a supplementary AI-assisted tool to identify and prioritize recent experimental and human studies. It was not used as a substitute for searches of conventional databases or as the sole basis for selecting studies. All publications identified using Elicit were independently assessed by the authors. Bibliographic information, study design, methods, main findings, and relevance to the review were verified against the original publications.

Priority was given to original research articles, randomized controlled trials, prospective cohort studies, pharmacokinetic studies, and recent experimental studies directly addressing polyphenol metabolism or cardiovascular outcomes. Systematic reviews and meta-analyses were included when they provided broader context or quantitative synthesis of the available evidence. Older publications were retained when they represented foundational studies or addressed questions not adequately covered by more recent research. As this is a critical narrative review, no formal risk-of-bias assessment or meta-analysis was performed.

## 2. Classification, Chemical Properties, Biological Activities and Dietary Sources of Polyphenols

Polyphenols represent a large and structurally diverse group of plant secondary metabolites characterized by the presence of one or more aromatic rings bearing multiple hydroxyl substituents. In a strict chemical sense, the term polyphenols generally refers to compounds containing multiple phenolic units, whereas simpler structures, such as phenolic acids, are more accurately described as phenolic compounds. However, nutritional and food-science literature commonly uses “dietary polyphenols” as an operational umbrella term that includes phenolic acids, flavonoids, stilbenes, lignans, and tannins. This broader convention is adopted throughout the present review.

**Figure 1:**
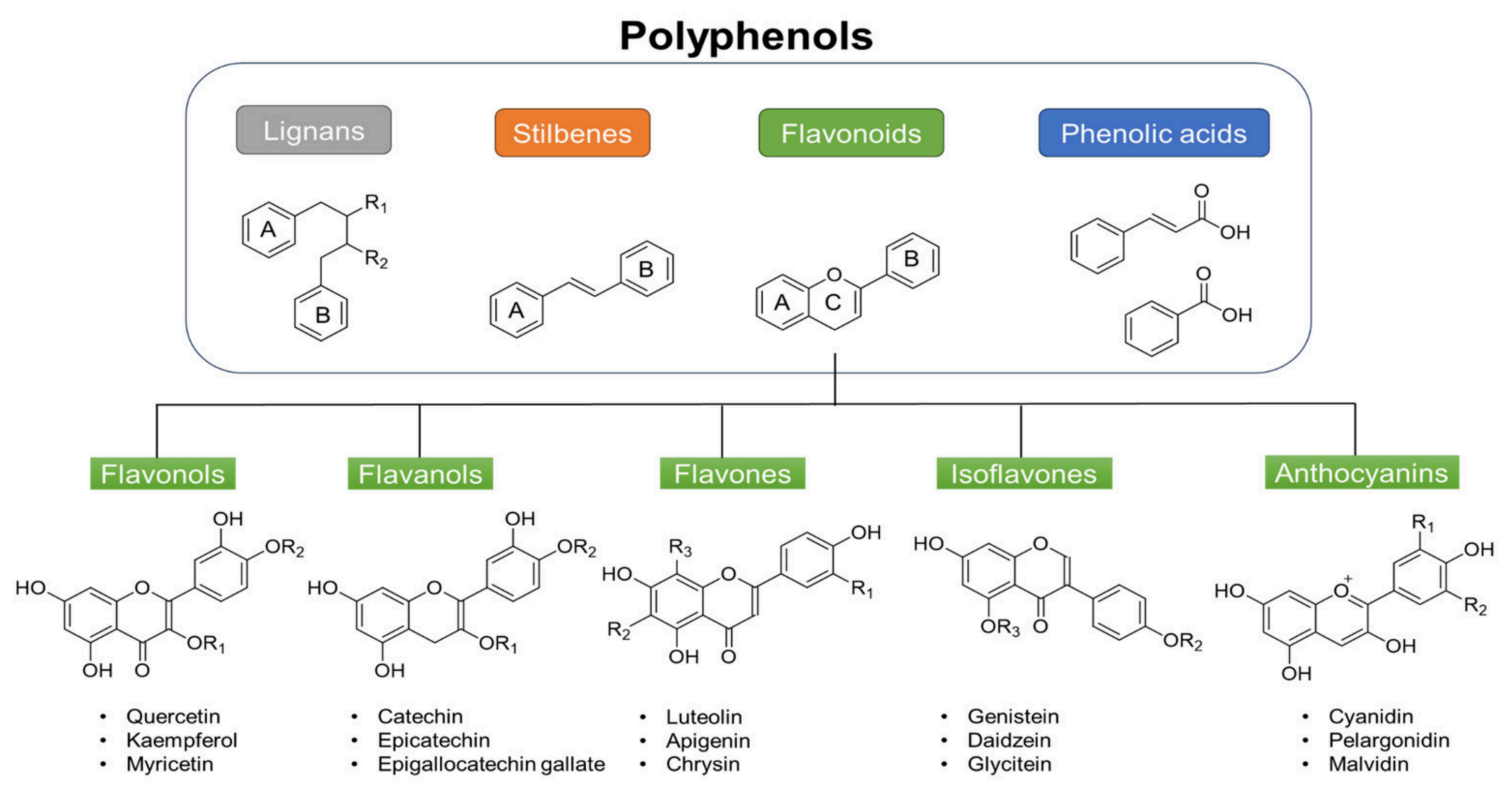
Simplified classification of the principal low-molecular-weight dietary phenolic classes discussed in this review. Tannins are described separately in the text.

More than 8000 plant phenolic compounds have been described, with substantial variation in molecular size, hydroxylation, glycosylation, esterification, and degree of polymerization. These structural features influence solubility, chemical stability, food-matrix interactions, intestinal absorption, host conjugation, microbial metabolism, systemic exposure, and biological activity. Figure 1 provides a simplified overview of the principal low-molecular-weight classes considered in this review, namely flavonoids, phenolic acids, stilbenes, and lignans. Oligomeric and polymeric compounds, particularly hydrolyzable and condensed tannins, are discussed separately in the text because their size and structural complexity result in distinct patterns of bioavailability and microbial transformation.

### 2.1. Flavonoids

Flavonoids constitute the largest and most extensively studied class of dietary polyphenols, accounting for approximately two-thirds of total polyphenol intake in Western diets. They share a common C_6_–C_3_–C_6_ carbon skeleton composed of two aromatic rings (A and B) linked via a heterocyclic oxygen-containing pyran ring (C) (Figure 1). Structural variations in hydroxylation patterns, degree of oxidation, saturation of the C ring, and glycosylation generate multiple subclasses, including flavonols, flavan-3-ols, flavones, flavanones, isoflavones, and anthocyanins. Flavan-3-ols occur as monomers, such as catechin and epicatechin, and as oligomeric or polymeric proanthocyanidins, also known as condensed tannins.

These structural differences strongly influence their chemical reactivity, stability, and biological activity. From a mechanistic perspective, flavonoids can act as hydrogen or electron donors, enabling scavenging of reactive oxygen species (ROS), as well as chelation of transition metal ions. Their antioxidant potential is particularly influenced by the presence of ortho-dihydroxyl groups in the B ring and conjugation across the C ring system. Beyond direct redox activity, flavonoids may exert pleiotropic biological effects, including anti-inflammatory, vasodilatory, antithrombotic, antipathogenic, and cardioprotective actions, largely mediated through modulation of intracellular signaling pathways rather than simple radical scavenging [18]. Tannins comprise oligomeric or polymeric phenolic compounds with limited absorption in their intact forms. Condensed tannins, or proanthocyanidins, are composed of flavan-3-ol units, whereas hydrolyzable tannins include gallotannins and ellagitannins containing gallic acid- or hexahydroxydiphenoyl-derived units. Their relatively high molecular mass favors passage to the colon, where proanthocyanidins and ellagitannin-derived ellagic acid are converted into lower-molecular-weight metabolites, including phenyl-γ-valerolactones and urolithins, respectively.

**Figure 2:**
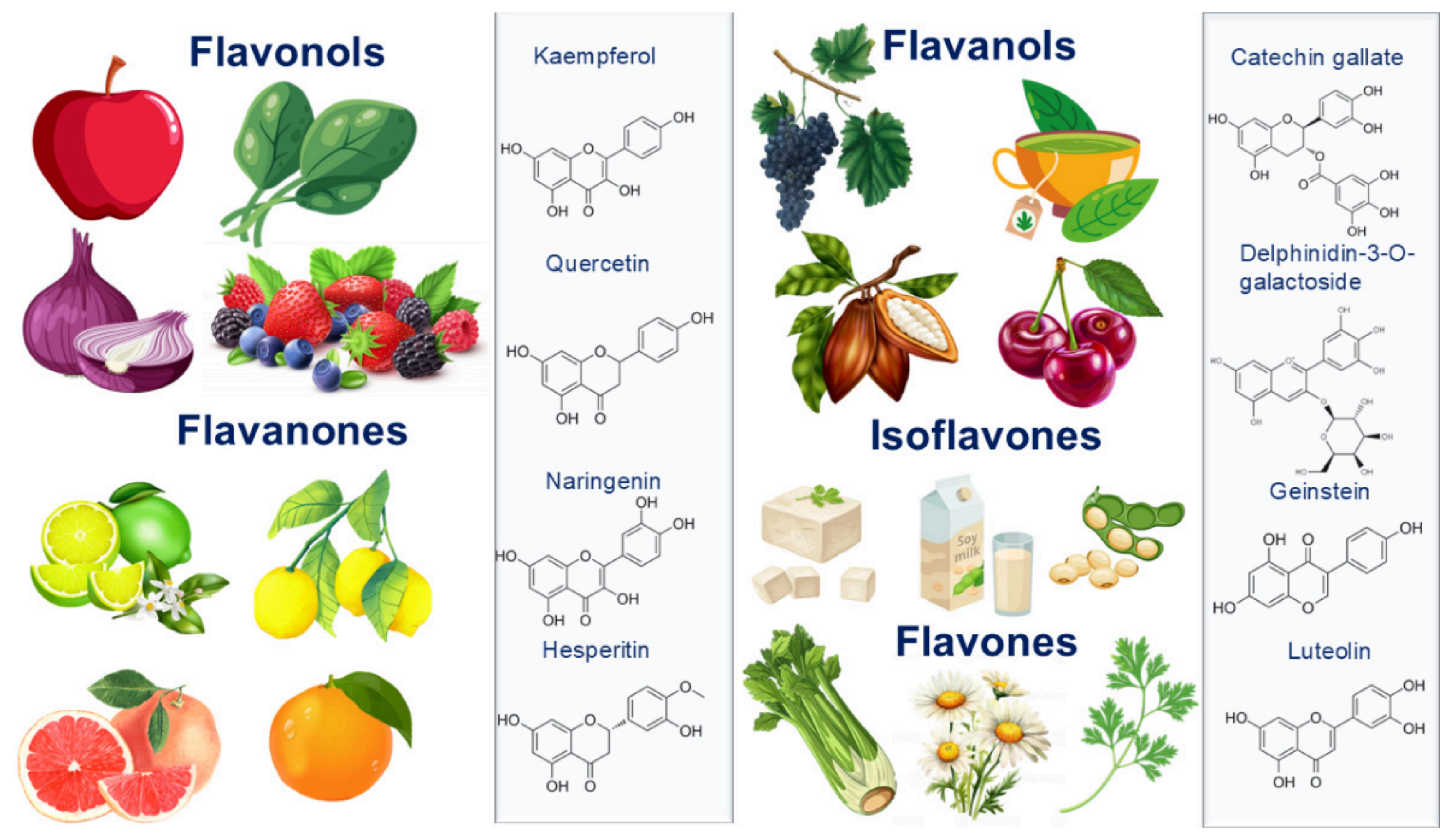
Major dietary sources of flavonoid subclasses.

Dietary sources are widely distributed among plant-based foods (Figure 2). Phenolic compounds are abundant in fruits, vegetables, cereals, legumes, beverages, and other plant-based foods, although their concentrations vary considerably among food sources. Hydroxybenzoic and hydroxycinnamic acids occur in berries, fruits, vegetables, cereals, coffee, and tea, with particularly high levels of hydroxycinnamic acids reported in blueberries, kiwi, cherries, artichoke, and coffee. Anthocyanins are abundant in deeply colored fruits and vegetables such as blackberries, black currants, blueberries, grapes, cherries, rhubarb, and red cabbage. At the same time, flavonols, mainly quercetin, kaempferol, and myricetin, are prominent in onions, kale, leeks, broccoli, berries, apples, and tomatoes. Flavones such as apigenin and luteolin are found mainly in parsley, celery, and peppers, whereas flavanones including hesperidin, naringenin, and eriodyctiol are characteristic of citrus fruits and juices. Isoflavones such as daidzein, genistein, and glycitein are particularly abundant in soy-based foods, while monomeric flavan-3-ols, including catechin and epicatechin, and oligomeric proanthocyanidins occur in chocolate, tea, grapes, berries, cherries, and red wine [19,20].

Phenolic compounds are not restricted to the edible portions of plants. Considerable amounts of phenolic compounds are also concentrated in by-products such as peels, skins, seeds, pomace, leaves, stems, husks, and other tissues generated during agricultural production and food processing. This biomass, traditionally considered waste or low-value by-products, can represent important sources of both extractable and non-extractable phenolics. Recent studies have systematically demonstrated that plant residue groups, including leaves and stalks, peels and husks, pulp and pomace, and seeds, contain characteristic phenolic profiles that can be targeted through selective extraction strategies [21,22,23,24,25]. Fruit-processing residues such as grape, apple, citrus, pomegranate, berry, and tomato pomaces, as well as grape skins and seeds, are particularly rich in flavonoids, anthocyanins, flavan-3-ols, and phenolic acids. Similarly, vegetable-processing residues, including onion and garlic skins, tomato peels and seeds, potato peels, broccoli stems and leaves, cabbage outer leaves, carrot residues, and beetroot by-products, contain substantial amounts of phenolic acids and flavonoids [26,27]. For example, chlorogenic acid and caffeic acid derivatives are frequently reported in potato, tomato, and vegetable residues, while quercetin and its glycosides are characteristic of onion skins. Citrus peels are particularly rich in flavanones such as hesperidin and naringin, whereas grape pomace and seeds contain abundant flavan-3-ols, anthocyanins, and phenolic acids [22,27].

Recent research has increasingly shifted from considering these materials as waste toward their use as secondary sources of dietary phenolics and high-value bioactive ingredients. Recent databases and reviews indicate that fruit-derived by-products currently dominate the literature on polyphenol recovery from agri-food residues, although vegetable by-products remain comparatively underexplored. In the PhInd database, fruit by-products accounted for 73.2% of the recorded entries, highlighting both their recognized potential and the need for greater investigation of vegetable residues [26]. Moreover, phenolics in these matrices occur not only in free or extractable forms but also as conjugated and non-extractable compounds associated with cell-wall components, making agro-food residues particularly interesting targets for advanced extraction and bioconversion strategies [28,29].

Thus, fruits and vegetables should be considered not only as dietary sources of phenolic compounds, but also as feedstocks for the recovery of phenolics from processing residues. The valorization of fruit and vegetable peels, seeds, pomaces, leaves, and stems can simultaneously reduce food-processing waste and provide sustainable sources of antioxidants and other bioactive compounds for functional foods, nutraceuticals, cosmetics, and other high-value applications [27,30]. Recent studies emphasize green extraction, including ultrasound-, microwave-, pulsed-electric-field-, enzyme-, and other intensified approaches, as promising strategies for recovering these compounds while supporting circular bioeconomy concepts [31,32,33].

### 2.2. Phenolic Acids

Phenolic acids are relatively simple phenolic compounds characterized by an aromatic ring bearing one or more hydroxyl groups and a carboxylic acid-containing substituent. Although they are not necessarily polyphenols in the strict structural sense, they are conventionally included within the broad nutritional category of dietary polyphenols. They are broadly divided into hydroxybenzoic and hydroxycinnamic acids (Figure 1), which differ in carbon skeleton length and substitution patterns. These compounds are commonly present in both free and conjugated forms (esters or glycosides), a feature that significantly influences their absorption, stability, and metabolic fate in the gastrointestinal tract. Hydroxybenzoic acid derivatives include gallic acid, which occurs in free and esterified forms, and ellagic acid, which is commonly released through the hydrolysis of ellagitannins found in berries, walnuts, and pomegranate. In contrast, hydroxycinnamic acids, including caffeic, ferulic, and para-coumaric acids, are more widely distributed in plant-based foods and represent the most abundant phenolic acids in the human diet. Functionally, phenolic acids exhibit antioxidant and anti-inflammatory properties, partly through inhibition of lipid peroxidation and modulation of inflammatory mediators such as cyclooxygenase-2 (COX-2) and pro-inflammatory cytokines. Notably, chlorogenic acid, a major caffeic acid derivative abundant in coffee, has been investigated in human intervention studies showing effects on early postprandial glucose and insulin responses, as well as on vascular endothelial function, suggesting potential relevance for glucose homeostasis and cardiometabolic risk modulation [34,35].

### 2.3. Stilbenes

Stilbenes are a relatively small but biologically potent class of polyphenols characterized by a 1,2-diphenylethylene backbone (Figure 1). Despite limited dietary abundance, they have attracted significant attention due to high bioactivity. The most extensively studied stilbene, resveratrol (3,5,4′-trihydroxystilbene), exists in cis- and trans- isomeric forms, with trans-resveratrol representing the predominant and most extensively studied biologically active isomer. Resveratrol may exert pleiotropic effects relevant to cardiovascular protection, including modulation of SIRT1-dependent mitochondrial biogenesis and endothelial nitric oxide synthase (eNOS)-related signaling, mechanisms that may contribute to improved vascular homeostasis and anti-atherogenic activity [36]. In humans, a one-year randomized, placebo-controlled trial showed that a resveratrol-rich grape supplement improved inflammatory and fibrinolytic biomarkers in patients at high cardiovascular risk, supporting its potential cardioprotective relevance [37]. Although resveratrol occurs in foods such as grapes, red wine, peanuts, and berries, population-based dietary assessments indicate that habitual intake is relatively low compared with more abundant dietary polyphenol classes [38].

### 2.4. Lignans

**Figure 3:**
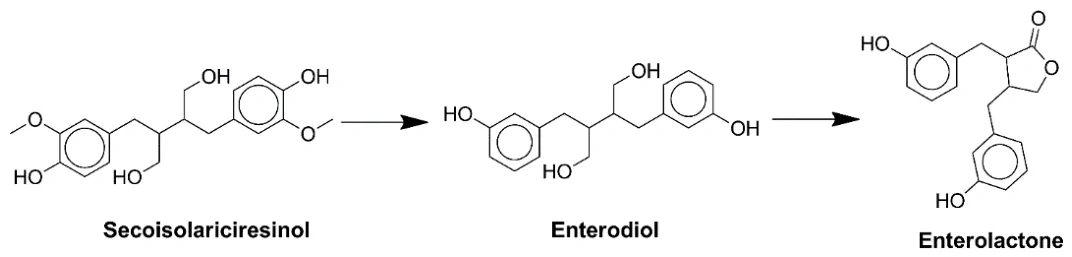
Microbial conversion of plant lignans into mammalian enterolignans. Secoisolariciresinol, a representative dietary lignan, is transformed by intestinal microbiota into enterodiol and subsequently into enterolactone, which are considered major bioactive enterolignan metabolites.

Lignans are diphenolic compounds formed through the oxidative coupling of two phenylpropanoid units. Although chemically stable, their biological activity depends heavily on intestinal microbial metabolism, which converts plant lignans into the bioactive enterolignans enterodiol and enterolactone (Figure 3) [39]. These metabolites exhibit weak estrogenic and anti-estrogenic properties through interaction with estrogen receptors and are therefore classified as phytoestrogens. In addition, lignans possess antioxidant and anti-inflammatory activities and may contribute to cardiometabolic health through modulation of lipid metabolism, insulin sensitivity, and hormonal balance [40]. Flaxseeds represent the richest dietary source of lignans, followed by sesame seeds, whole grains (notably rye, oats, and wheat), legumes, and certain fruits and vegetables such as berries and broccoli. Importantly, interindividual variability in gut-microbiota composition plays a decisive role in the conversion efficiency of lignans to enterolignans, thereby influencing their biological effects.

### 2.5. Dietary Intake and Food Pattern Variability

Total dietary polyphenol intake exhibits substantial interindividual and interpopulation variability, reflecting differences in dietary habits, food choices, and availability of plant-derived foods. Estimated intakes may range from less than 200 mg/day in diets poor in fruits, vegetables, beverages, and other plant-based foods to more than 1500–2000 mg/day in polyphenol-rich dietary patterns. In the European Prospective Investigation into Cancer and Nutrition (EPIC) cohort, mean total polyphenol intake generally ranged from approximately 700–800 mg/day in Mediterranean population to over 1200–1500 mg/day in Northern and Central European populations, with coffee and tea consumption accounting for much of this geographical variation [41,42].

At the level of individual polyphenol classes, phenolic acids and flavonoids are the dominant contributors to total polyphenol intake. Phenolic acids typically account for 50–57% of total polyphenol exposure and provide 500–1000 mg/day, largely through hydroxycinnamic acids originating from coffee consumption. Flavonoids represent the second major class, contributing about 200–600 mg/day depending on dietary habits. In contrast, lignans and stilbenes contribute comparatively small amounts, generally below 10 mg/day and about 3 mg/day, respectively, within European populations [41,43].

The principal dietary sources of polyphenols differ across populations but are dominated by a relatively small number of foods and beverages. In Western countries, coffee is typically the main contributor, supplying substantial quantities of hydroxycinnamic acids and, in heavy consumers, contributing approximately 400–800 mg/day. Tea represents another major source, providing flavanols and flavonols and often contributing an additional 100–300 mg/day depending on consumption frequency [19,41]. Fruits, particularly berries and citrus fruits, together with cocoa products and red wine, further contribute flavonoids, phenolic acids, and stilbenes such as resveratrol. Consequently, beverages frequently account for a greater proportion of total polyphenol intake than solid foods in many European populations [42,44,45].

Dietary patterns strongly influence both the quantity and diversity of polyphenol exposure. The Mediterranean diet is among the best-characterized dietary models rich in polyphenols and has been associated with reduced cardiovascular risk. Key contributors include extra-virgin olive oil, which provides hydroxytyrosol, tyrosol, and oleuropein derivatives, fruits (e.g., grapes, pomegranates, citrus fruits, and figs), vegetables (tomatoes, onions, and leafy greens), legumes, whole grains, nuts, herbs and spices, together with moderate red wine consumption in some populations. This dietary pattern promotes exposure to a broad spectrum of structurally diverse polyphenols, which may exert complementary biological actions through multiple overlapping mechanisms [46].

In contrast, Western dietary patterns characterized by high consumption of refined carbohydrates and ultra-processed foods and lower intake of fruits, vegetables, legumes, and other plant-derived foods are associated with substantially lower amounts and significantly reduced structural diversity of dietary polyphenols. Such dietary profiles are frequently associated with less favorable cardiometabolic outcomes, although these relationships remain influenced by numerous dietary, behavioral, and environmental factors [46].

Despite growing interest in the health effects of polyphenols, accurate assessment of dietary intake remains challenging. Polyphenol concentrations vary considerably according to plant genotype, geographical origin, environmental conditions, agricultural practices, ripening stage, storage conditions, food processing, and culinary preparation. Although databases such as Phenol-Explorer and the USDA Flavonoid Database provide improved exposure assessment, limitations in food coverage, analytical standardization, and database harmonization continue to introduce uncertainty into intake estimates and complicate comparisons among studies. Increasing evidence suggests that integration of dietary assessment tools with validated urinary or plasma biomarkers may improve the accuracy and reproducibility of polyphenol exposure measurements in epidemiological and clinical investigations [47,48,49].

## 3. Bioavailability and Metabolic Fate of Dietary Polyphenols: Implications for Cardiovascular Bioactivity

The potential cardiovascular benefits of dietary polyphenols cannot be considered independent from their bioaccessibility, bioavailability, and metabolic fate. Bioaccessibility refers to the fraction of an ingested compound released from the food matrix during gastrointestinal digestion and made available for intestinal absorption. Bioavailability, in contrast, describes the fraction of ingested compound, or its metabolites, that reaches the systemic circulation and is potentially available for tissue distribution. Actual biological activity further depends on tissue exposure, intracellular availability, molecular target engagement, and the activity of the circulating chemical species. Although bioaccessibility is an important prerequisite for subsequent absorption, the present section focuses primarily on bioavailability and the metabolic fate of polyphenols after ingestion.

**Figure 4:**
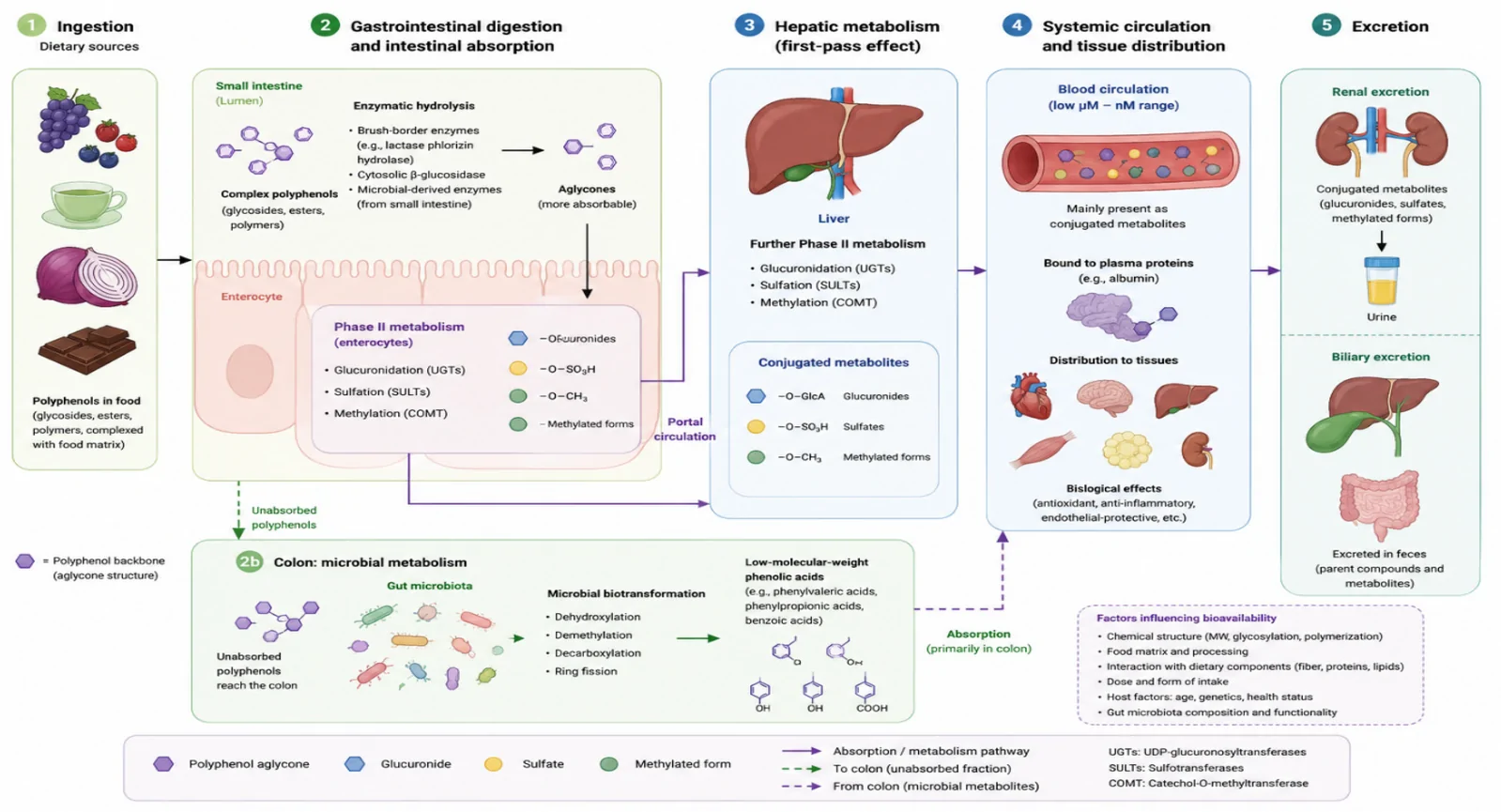
Schematic overview of the absorption, metabolism, and systemic fate of dietary polyphenols.

Following ingestion, polyphenols pass through a sequence of food-matrix release, gastrointestinal transformation, intestinal absorption, presystemic metabolism, microbial biotransformation, hepatic transformation, systemic distribution, and elimination. After release from the food matrix, they generally follow two complementary metabolic routes. Compounds absorbed in the small intestine may undergo extensive enterocyte and hepatic conjugation before entering the systemic circulation. In contrast, compounds that escape upper-gastrointestinal absorption reach the colon, where the gut microbiota converts them into lower-molecular-weight metabolites that may subsequently be absorbed, further transformed by the host, and distributed systemically. An integrated overview of the absorption, host and microbial metabolism, systemic distribution, and elimination of dietary polyphenols is presented in Figure 4.

Consequently, the chemical species present in the plasma and ultimately available to peripheral tissues may differ substantially from the parent compounds originally consumed. Bioavailability should therefore be considered a central determinant of polyphenol bioactivity, particularly when translating mechanistic and in vitro findings to cardiovascular effects in vivo [4,19,50,51]. Detailed quantitative comparisons of the bioavailability and pharmacokinetics of major dietary polyphenols are available in specialized reviews [19,50]. The present section instead focuses on representative human data that illustrate the distinction between ingested parent compounds, circulating conjugates, microbiota-derived metabolites, and tissue-relevant exposure.

### 3.1. Gastrointestinal Release, Stability, and Intestinal Absorption

Before absorption can occur, polyphenols must be released from the food matrix and remain sufficiently stable during gastric and intestinal digestion. These processes are influenced by food composition and processing, interactions with proteins, lipids, carbohydrates, and minerals, gastrointestinal pH, digestive enzymes, and the physicochemical properties of the individual compound. Bioaccessibility therefore represents an essential, although not sufficient, prerequisite for systemic exposure [19,50].

The extent and rate of intestinal absorption vary considerably among polyphenol classes. Major structural determinants include molecular size, glycosylation pattern, degree of polymerization, lipophilicity, solubility, and chemical stability. The food matrix and intestinal transport mechanisms further influence epithelial uptake. Aglycones, monoglycosides, and diglycosides can consequently display markedly different patterns of gastrointestinal release and intestinal absorption [19,52].

Hydrolysis of polyphenol glycosides may occur in the intestinal lumen, at the brush border through enzymes such as lactase-phlorizin hydrolase, or within enterocytes after the uptake and cleavage of intact glycosides by cytosolic β-glucosidases. The efficiency of these processes depends on the polyphenol structure and the identity and position of the sugar moiety. The resulting aglycones may cross epithelial membranes by passive diffusion or interact with carrier-mediated transport and efflux systems. Glycosides that are neither hydrolyzed or absorbed in the small intestine may reach the colon, where they undergo microbial deglycosylation and further catabolism. Intestinal absorption therefore involves multiple processes rather than passive diffusion alone. For example, some flavonoid glycosides interact with intestinal hexose-transport systems, including sodium–glucose cotransporter 1 and glucose transporter 2, although the contribution of these transporters varies among compounds and experimental models [52].

Following epithelial uptake, polyphenols may be transported into the portal circulation, retained temporarily within enterocytes, or returned to the intestinal lumen through apical efflux transporters. The proportion reaching portal blood thus depends on the combined effects of gastrointestinal stability, epithelial uptake, intracellular metabolism, basolateral export, and transporter-mediated apical efflux.

### 3.2. Intestinal Presystemic Metabolism and Transport

Once taken up by intestinal epithelial cells, polyphenols can undergo extensive presystemic metabolism. Oxidation and reduction reactions may occur in enterocytes and hepatocytes, although their contribution is generally less prominent than that of conjugative metabolism for many polyphenols. Importantly, hydrolysis of dietary glycosides may occur independently of classical Phase I metabolism and at several locations along the gastrointestinal tract.

Phase II reactions, particularly glucuronidation, sulfation, and methylation, take place in both enterocytes and hepatocytes. These reactions are catalyzed principally by UDP-glucuronosyltransferases, sulfotransferases, and catechol-O-methyltransferase and generate products with altered polarity, protein binding, transporter affinity, and elimination kinetics. Phase II conjugation does not necessarily require prior Phase I metabolism. Polyphenols can therefore be conjugated during intestinal first-pass metabolism and undergo further transformation during hepatic first-pass metabolism [19,53,54].

Transporter-mediated disposition further determines intestinal availability. Polyphenols and their conjugates may be exported across the basolateral membrane into portal blood or returned across the apical membrane into the intestinal lumen. Human intestinal models have demonstrated methylation and sulfation of epigallocatechin gallate, together with apical efflux mediated by multidrug resistance-associated protein 2 [55]. Intestinal bioavailability therefore reflects the net balance among epithelial uptake, intracellular metabolism, and transporter-mediated efflux rather than passive diffusion alone [52,55].

These processes are particularly relevant when evaluating cardiovascular mechanisms. Many experimental studies investigate the effects of unconjugated polyphenols at concentrations that may substantially exceed those measured in human plasma following dietary exposure. Circulating polyphenol-derived compounds are more commonly present as glucuronidated, sulfated, methylated, or microbiota-derived metabolites [56]. These products may differ from the corresponding parent compounds in chemical stability, cellular uptake, biomolecular interactions, tissue distribution, and biological activity. Therefore, the cardiovascular relevance of an individual polyphenol should not be inferred solely from the concentration or activity of its unconjugated form.

### 3.3. Colonic Microbial Transformation and Secondary Absorption

Polyphenols that are not absorbed in the stomach or small intestine follow a complementary metabolic route through the colon. This fraction may be substantial for oligomeric and polymeric compounds, including proanthocyanidins and hydrolyzable tannins, as well as for poorly soluble aglycones, glycosides inefficiently hydrolyzed by host enzymes, and compounds strongly associated with the food matrix [57].

In the colon, dietary polyphenols become substrates for bacterial glucosidases, glucuronidases, esterases, reductases, and ring-cleaving enzymes. These enzyme systems catalyze deglycosylation, deconjugation, demethylation, dehydroxylation, decarboxylation, hydrogenation, and aromatic-ring cleavage. The resulting products include phenylacetic, phenylpropionic, phenylvaleric, hydroxybenzoic, and other phenolic acids, as well as phenyl-γ-valerolactones and urolithins. These lower-molecular-weight products can be more readily absorbed than their dietary precursors and may contribute substantially to systemic exposure.

The extent and profile of microbial metabolism depend on both the chemical structure of the substrate and the metabolic capacity of the individual microbiota. For example, recent work with cranberry proanthocyanidins demonstrated structure-dependent microbial metabolism, with differences related to interflavan structure and degree of polymerization [57]. Similarly, human gut-microbiota experiments have identified diverse low-molecular-weight bacterial catabolites following exposure to cocoa flavanols, ellagic acid, naringenin, and phloroglucinol [58,59].

After absorption from the colon, microbial metabolites enter the portal circulation and may undergo additional glucuronidation, sulfation, or methylation in the intestinal epithelium and liver. Compounds detected in plasma can therefore represent a mixture of residual parent compounds, primary host conjugates, microbial catabolites, and host conjugates of microbial products [56,60]. Gut-microbial metabolism should consequently be regarded as an integral component of polyphenol pharmacokinetics rather than simply as a mechanism of compound degradation.

Human microbiota studies demonstrate significant donor-dependent differences in the rate, composition, and abundance of metabolites generated from the same polyphenol precursors [61,62]. These differences provide a plausible explanation for why individuals consuming comparable amounts of a polyphenol-rich food may achieve markedly different systemic exposures.

**Figure 5:**
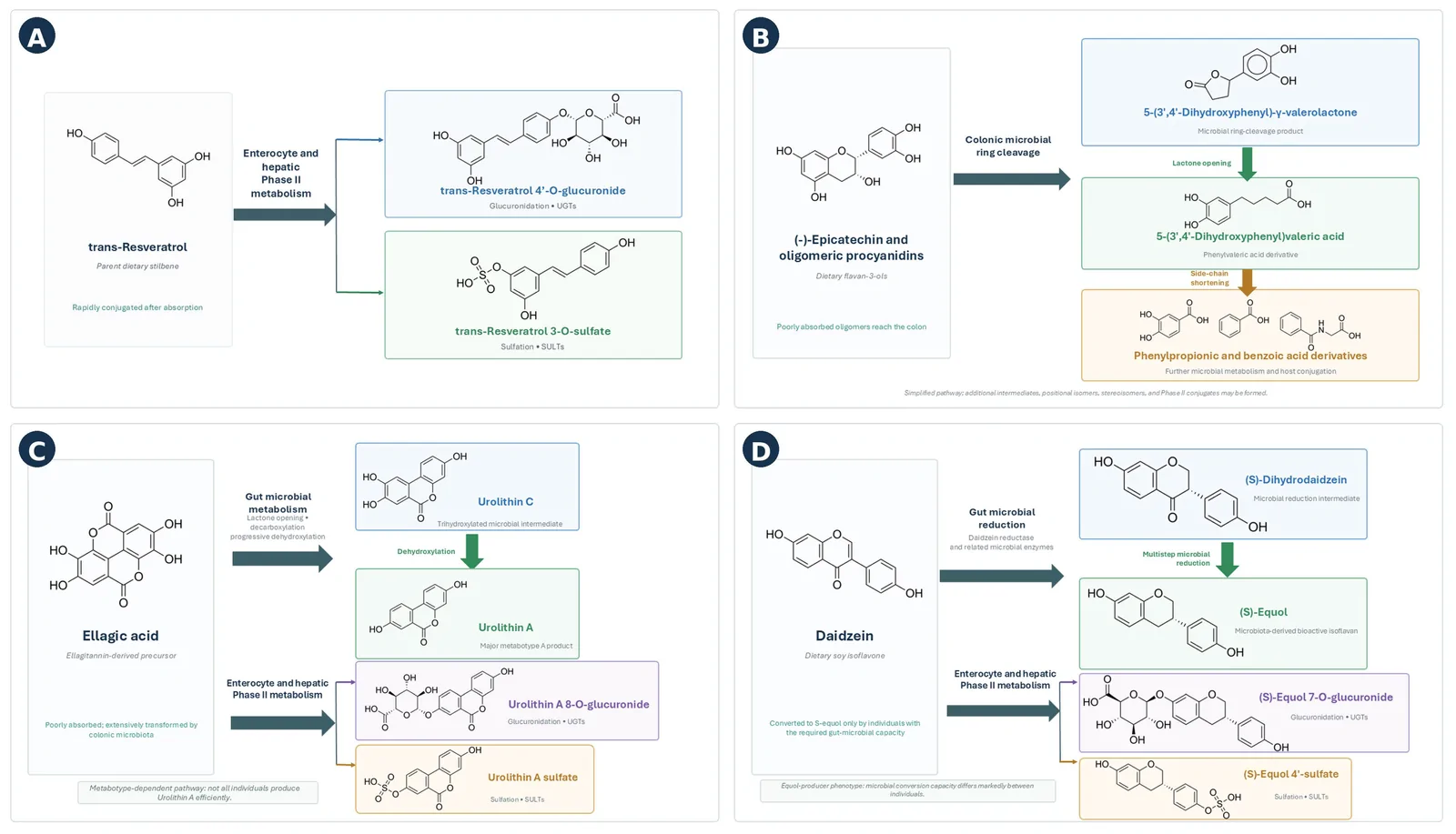
Representative host- and microbiota-mediated metabolic pathways of dietary polyphenols relevant to cardiovascular bioactivity. The figure distinguishes ingested parent compounds, primary host-derived conjugates, gut microbiota-derived metabolites, and secondary host conjugates according to their positions within the metabolic pathways. (**A**) Trans-resveratrol undergoes rapid intestinal and hepatic glucuronidation and sulfation, resulting in low systemic exposure to the unchanged parent compound. (**B**) Flavan-3-ols and oligomeric procyanidins that reach the colon are converted into phenyl-γ-valerolactones and subsequently into lower-molecular-weight phenolic acids. (**C**) Hydrolyzable ellagitannins release ellagic acid, which is transformed by the gut microbiota through sequential dehydroxylation into urolithins that circulate mainly as Phase II conjugates. (**D**) Daidzein is converted to (*S*)-equol only in individuals harboring the required microbial metabolic capacity. The pathways are simplified and show representative metabolites; additional positional isomers, intermediate products, and conjugated derivatives may also be formed.

Representative metabolic pathways further illustrate the diversity of polyphenol transformations (Figure 5). Trans-resveratrol undergoes rapid intestinal and hepatic glucuronidation and sulfation, resulting in low systemic exposure to the unchanged parent compound [63,64]. Flavan-3-ols that reach the colon undergo microbial ring cleavage to form phenyl-γ-valerolactones and phenylvaleric acids, followed by further degradation to smaller phenolic and host–microbial co-metabolites [65,66]. Ellagic acid is converted into urolithins through microbiota-mediated lactone opening, decarboxylation, and dehydroxylation [67,68], whereas daidzein can be reduced to (*S*)-equol only in individuals harboring the required microbial enzymatic capacity [69]. Following absorption, urolithins and equol undergo further Phase II conjugation and circulate predominantly as glucuronide and sulfate derivatives [60].

### 3.4. Hepatic Metabolism, Systemic Distribution, and Elimination

Polyphenols absorbed in the small intestine and microbial metabolites absorbed from the colon enter the portal circulation and undergo further processing in the liver. Hepatic glucuronidation, sulfation, methylation, oxidation, and reduction can modify their polarity, plasma persistence, protein binding, transporter recognition, tissue distribution, and routes of elimination. Human intervention studies show that circulating and urinary polyphenol-derived compounds are frequently detected as Phase II conjugates rather than as unconjugated aglycones [60,70].

Human pharmacokinetic studies commonly reveal at least two temporally distinct components of polyphenol exposure. Compounds absorbed in the upper-gastrointestinal tract and their enterocyte- or liver-derived conjugates often appear in plasma within the first few hours after ingestion. On the other hand, metabolites generated through colonic microbial transformation commonly appear later and may remain in circulation for longer periods. Although the timing and magnitude of these profiles are highly compound-specific, circulating concentrations are generally in the nanomolar-to-low-micromolar range, while unconjugated parent compounds are often undetectable or present only transiently [4,19,50]. Following ingestion of ^13^C-labelled spinach, labelled methoxyflavonol glucuronides and sulfates were detected in plasma, with measurable interindividual differences in their pharmacokinetic profiles [70]. Similarly, after four weeks of pecan consumption, urolithin-related metabolites were detected predominantly as glucuronide conjugates in plasma, demonstrating that microbiota-derived metabolites can contribute substantially to systemic exposure following repeated dietary exposure [60].

Resveratrol provides a clear example of the distinction between intestinal absorption and systemic exposure to an unchanged parent compound. Following a 25 mg oral dose in humans, at least 70% of resveratrol was absorbed, and peak plasma concentrations of total resveratrol-derived species reached approximately 2 μM. However, unchanged resveratrol remained below 5 ng/mL because of rapid and extensive glucuronidation and sulfation [63]. Measurements of total resveratrol-derived species may therefore provide a more informative estimate of systemic exposure than measurements of unconjugated resveratrol alone [71].

Following hepatic processing, polyphenol-derived compounds may enter the systemic circulation, undergo renal elimination, or be excreted in bile (Figure 4). Conjugates returned to the intestinal lumen through biliary excretion may be hydrolyzed by microbial glucuronidases, sulfatases, or other enzymes, allowing further microbial transformation or reabsorption of released products. For selected compounds, this enterohepatic cycling may prolong their apparent residence time or contribute to secondary plasma peaks, although its quantitative importance is compound-specific [50,54].

Systemic exposure does not, however, necessarily correspond to exposure at cardiovascular target tissues. Tissue distribution depends on molecular structure, plasma protein binding, transporter recognition, conjugation state, local deconjugation, and tissue-specific uptake. The gastrointestinal tract and liver may experience greater and more prolonged exposure than many peripheral tissues, while access to vascular, cardiac, renal, or adipose tissues may differ considerably among individual metabolites. Therefore, the detection of a metabolite in plasma does not demonstrate that it reaches a cardiovascular target tissue at a biologically relevant concentration. Plasma concentration should be regarded as one measure of systemic exposure rather than as direct evidence of tissue-level bioactivity.

### 3.5. Interindividual Variability and Metabolic Phenotypes

Interindividual variability represents a major determinant of polyphenol bioavailability and biological response. Gut-microbiota composition and metabolic activity can markedly influence the rate and profile of polyphenol biotransformation, while host characteristics, dietary context, disease status, medication use, and genetic factors can influence the rate, extent, and profile of polyphenol metabolism [46,47,56].

Fecal-fermentation studies have demonstrated donor-specific metabolite profiles following exposure to black-tea, red-wine, grape-juice polyphenols, and epicatechin. The variability is not only quantitative but also qualitative, as individuals may generate different metabolite sets or different relative proportions of the same metabolites [61]. These differences may help explain why individuals consuming comparable amounts of polyphenol-rich foods achieve different systemic exposures.

Urolithin metabotypes provide a well-characterized example. Individuals can be classified according to their capacity to generate urolithin A, isourolithin A, urolithin B, or no final urolithins after ellagitannin or ellagic acid exposure. In the MaPLE trial, urolithin metabotypes were associated with differences in microbial and metabolomic responses to a polyphenol-rich dietary intervention [72]. Daidzein-to-equol conversion represents another categorical microbial phenotype, since only individuals possessing the required microbial enzymatic network efficiently generate (*S*)-equol [68]. These phenotypes illustrate how the same dietary precursor may generate qualitatively different systemic exposures.

From a cardiovascular perspective, this variability may be particularly important because individuals consuming similar amounts of a polyphenol-rich food may experience different systemic exposures and consequently different biological responses. Future clinical studies should therefore consider combining dietary exposure with plasma or urinary metabolite profiling and, where appropriate, characterization of gut-microbial metabolic phenotypes [44,72,73].

### 3.6. Implications for Cardiovascular Bioactivity and Clinical Translation

Cardiovascular exposure following polyphenol intake comprises a time-dependent mixture of residual parent compounds, host-derived conjugates, microbial catabolites, and secondary conjugates. Consequently, low plasma concentrations of an intact polyphenol do not exclude biological relevance, but detection of a metabolite in plasma does not establish tissue delivery, target engagement, or cardiovascular efficacy. Mechanistic studies should therefore use chemical forms, concentrations, and exposure times consistent with human pharmacokinetic data. The cardiovascular effects associated with these exposures are considered in Section 4.

## 4. Mechanisms Linking Polyphenols and Their Metabolites to Cardiovascular Protection

The cardiovascular effects associated with polyphenol-rich foods cannot be attributed to a single universal mechanism or chemical entity. Mechanistic evidence should be interpreted according to the experimental level, as findings from cell-based studies, animal experiments, and human studies do not provide equivalent evidence. In addition, concentrations used in some in vitro studies may exceed those achievable in human plasma. Parent compounds, host-derived conjugates, microbial metabolites, and other components of the food matrix may act at different anatomical sites and time points. Their effects may involve vascular endothelial function, inflammatory and redox-sensitive signaling, mitochondrial homeostasis, lipid metabolism, platelet activity, and the regulation of vascular tone. However, the strength of evidence differs markedly among these mechanisms. Many proposed molecular targets originate from cell or animal models, whereas human evidence more commonly concerns functional outcomes such as flow-mediated dilation, blood pressure, plasma lipids, or metabolite-associated risk signatures.

### 4.1. Endothelial Function and Nitric Oxide-Dependent Vascular Responses

Endothelial dysfunction is characterized by impaired vasodilatation, reduced nitric oxide availability, oxidative stress, inflammatory activation, and altered interactions among endothelial cells, platelets, and leukocytes. Polyphenol-rich foods, particularly cocoa, tea, berries, apples, and grapes, have been associated with improved endothelial function, although the chemical species responsible for these effects are not always identified [5,16,35].

Flavan-3-ols provide some of the strongest evidence for vascular effects. In experimental systems, epicatechin activates endothelial nitric oxide synthase. Human interventions with flavan-3-ol-rich foods have also frequently reported improvements in flow-mediated dilation. A recent meta-analysis of 145 randomized comparisons involving more than 5000 participants found that both acute and repeated flavan-3-ol intake improved flow-mediated dilation and produced modest reductions in blood pressure. However, substantial heterogeneity remained, and the overall strength of the evidence was considered moderate rather than conclusive [74].

The biologically relevant vascular exposure may not be limited to the ingested flavan-3-ols. Gut microbiota-derived flavanol metabolites, including hydroxyphenylacetic, hydroxybenzoic, and hydroxyphenylpropionic acid derivatives, have stimulated nitric oxide-related signaling and protected human endothelial cells against oxidative injury in experimental models [75]. These findings support the biological plausibility of metabolite-mediated vascular effects, although they do not establish that any single metabolite is responsible for the response to a flavan-3-ol-rich food.

Human intervention studies provide additional evidence linking circulating metabolites with vascular responses. After consumption of ellagitannin-rich raspberries, improvements in flow-mediated dilation correlated with plasma urolithin A conjugates [76]. In a randomized crossover trial, an anthocyanin-rich blackcurrant beverage improved postprandial flow-mediated dilation, while selected circulating metabolites predicted variation in vascular and blood-pressure responses [77]. These findings suggest that host- and microbiota-derived metabolites may contribute to the observed effects, rather than the responses being mediated solely by the ingested parent anthocyanins.

These studies should nevertheless be interpreted with caution. Correlations between circulating metabolites and vascular responses are not straightforward, since multiple co-occurring metabolites may reflect the same dietary exposure or metabolic pathway. Further studies using physiologically relevant metabolite mixtures, direct measures of molecular target engagement, and sampling schedules that capture both early and delayed metabolite exposure are needed to identify the chemical species responsible for the observed vascular effects.

### 4.2. Redox-Sensitive and Inflammatory Signaling

The older model of polyphenol activity emphasized direct scavenging of reactive oxygen species. This mechanism is unlikely to explain most systemic effects at dietary achievable concentrations. Current evidence more strongly supports modulation of redox-sensitive signaling, endogenous antioxidant defense, mitochondrial function, inflammatory gene expression, and enzyme systems involved in reactive oxygen species production [4,18].

In endothelial and immune-cell models, polyphenols and selected metabolites can influence pathways involving nuclear factor-κB, mitogen-activated protein kinases, NADPH oxidases, heme oxygenase-1, and nitric oxide-dependent signaling. Protocatechuic and vanillic acids, which can arise during anthocyanin metabolism, have modulated endothelial oxidative stress and pathways associated with vascular homeostasis [78]. Microbial flavanol metabolites have likewise reduced oxidative injury and supported nitric oxide-related signaling in human endothelial cells [75].

The evidence does not justify treating all glucuronides, sulfates, or microbial phenolic acids as biologically interchangeable. Conjugation can reduce, preserve, redirect, or occasionally enable biological activity depending on the metabolite, target, and experimental system. Local deconjugation may also regenerate aglycones in particular inflammatory microenvironments, but the quantitative contribution of this mechanism in human cardiovascular tissues remains uncertain.

The blackcurrant intervention provides relevant human support for links among dietary polyphenols, metabolites, vascular function, platelet reactivity, and low-grade inflammatory responses. Nevertheless, the small sample size and acute postprandial design mean that these findings should be regarded as mechanistic evidence rather than proof of long-term cardiovascular risk reduction [77].

### 4.3. Mitochondrial Quality Control and Cellular Energy Metabolism

Mitochondrial dysfunction contributes to endothelial impairment, cardiomyocyte energetic failure, oxidative stress, insulin resistance, and age-related cardiovascular decline. Polyphenols have been associated experimentally with mitochondrial biogenesis, mitophagy, respiratory efficiency, and stress resistance, although many studies use parent compounds at concentrations exceeding normal dietary exposure [9,36].

Urolithin A provides a particularly relevant metabolite-centered example. In experimental models of cardiac ageing and heart failure, urolithin A improved mitochondrial ultrastructure, mitophagy-associated processes, and systolic or diastolic cardiac function. In the human component of the same investigation, four months of urolithin A supplementation reduced plasma ceramides associated with cardiovascular risk [79]. These human results concern biomarkers rather than clinical cardiovascular events and should not be presented as demonstrated cardioprotection in humans.

The urolithin A findings also require distinction between endogenous microbial production after ellagitannin consumption and administration of a standardized postbiotic supplement. Dietary formation depends on an individual’s capacity to produce urolithins through gut-microbial metabolism, whereas direct supplementation bypasses the need for microbial conversion. These exposures cannot be assumed to produce identical kinetics, tissue distributions, or biological effects.

### 4.4. Lipid Metabolism and Lipoprotein-Related Effects

Polyphenol-rich foods and extracts can influence plasma lipids, intestinal lipid handling, hepatic lipid metabolism, and lipoprotein turnover. Proposed mechanisms include altered cholesterol incorporation into mixed micelles, changes in intestinal cholesterol transport, modulation of hepatic lipogenesis and fatty-acid oxidation, and altered expression or degradation of proteins involved in lipoprotein assembly. Most detailed molecular pathways, however, are derived from experimental systems and should not be presented as universally established human mechanisms.

Meta-analyses indicate that green tea and resveratrol interventions may produce modest changes in selected lipid variables, but effects vary with dose, intervention form, baseline metabolic status, and study duration [80,81]. Whole-food and purified-extract interventions should be distinguished because they differ in polyphenol dose, food matrix, co-nutrients, and safety profile [82].

Cocoa-derived metabolites provide a possible link between microbial polyphenol metabolism and lipid responses. Following cocoa consumption, phenyl-γ-valerolactone and epicatechin-derived metabolites were associated with changes in HDL-cholesterol and other cardiometabolic variables [83]. Such associations are hypothesis-generating because the metabolites may represent biomarkers of exposure rather than independent causal effectors.

Accordingly, lipid-related claims should be framed at the level of observed human outcomes and plausible mechanisms rather than assigning direct control of individual hepatic proteins to dietary polyphenols without compound-specific and physiologically relevant evidence.

### 4.5. Blood Pressure and Vascular Tone

Polyphenol-rich interventions may influence blood pressure through a combination of endothelial nitric oxide signaling, changes in arterial stiffness, vascular smooth-muscle function, renal handling, and neurohumoral regulation. Proposed targets include angiotensin-converting enzyme, endothelin signaling, calcium handling, potassium channels, and oxidative pathways, but direct evidence at nutritionally achievable systemic concentrations is uneven.

The strongest human data concern flavan-3-ol-rich foods. Recent synthesis of randomized trials indicates average reductions of approximately 2–4 mmHg in office or ambulatory systolic blood pressure, with larger effects among individuals with elevated baseline blood pressure. Flow-mediated dilation improved independently of the blood-pressure response, suggesting that endothelial effects and blood-pressure changes are related but not identical outcomes [74]. Consistent with these data, higher flavan-3-ol intake was associated with lower blood pressure in the EPIC-Norfolk cohort. However, the observational study design does not establish a causal relationship [84].

Acute beverage studies also show that vascular responses can be associated with circulating phenolic metabolites rather than detectable parent compounds. In the blackcurrant trial, selected metabolites were associated with flow-mediated dilation and blood-pressure variables, but the study was not designed to prove that the metabolites directly caused the observed effects [77].

### 4.6. Platelet Function and Hemostatic Pathways

Platelet activation and thrombus formation contribute directly to myocardial infarction and ischemic stroke. Polyphenols may influence platelet aggregation, activation-marker expression, microparticle release, thromboxane-related signaling, and interactions between platelets and the vascular endothelium. However, available evidence is dominated by in vitro, ex vivo, and short-term dietary studies.

The anthocyanin-rich blackcurrant trial assessed collagen- and ADP-induced platelet aggregation alongside endothelial function and metabolite profiles. The intervention improved postprandial vascular function and affected platelet-related outcomes, providing useful human mechanistic evidence but not evidence in humans but not evidence of reduced clinical thrombosis [77]. In an experimental study, a phenolic-rich black sorghum extract also modulated platelet activation and platelet-derived microparticle release [85]. However, because black sorghum extract contains multiple polyphenols and other constituents, the study could not identify the chemical species responsible for these effects or establish clinical antithrombotic efficacy. Until larger and longer human trials are available, the effects of polyphenols on platelet function should be regarded as a possible contributing mechanism rather than as an established clinical action comparable to pharmacological antiplatelet therapy.

### 4.7. Cardiovascular Relevance of Circulating and Microbial Metabolites

In interpreting metabolite-related evidence, three levels should be distinguished: detection as a biomarker of exposure, association with a biological response, and demonstration of a causal effect at a physiologically relevant concentration. Most available human evidence concerns the first two levels.

Evidence linking polyphenol-derived metabolites directly to cardiovascular outcomes remains heterogeneous. In the PREDIMED analysis, 62 urinary phenolic metabolites were quantified in 1180 participants, including 653 who subsequently developed cardiovascular disease. An eight-metabolite signature, comprising catechol, vanillin glucuronide, urolithin A metabolites, m-coumaric acid, naringenin glucuronide, hydroxytyrosol, protocatechuic acid and hydroxytyrosol sulfate, was associated with greater adherence to the Mediterranean diet and a lower incidence of cardiovascular disease. Participants in the highest quartile of the metabolite signature had approximately 52% lower cardiovascular risk than those in the lowest quartile [86]. However, the signature may reflect dietary quality, microbial metabolism, host processing, and correlated lifestyle factors and should therefore be viewed as an integrated exposure marker rather than proof that each metabolite independently reduces cardiovascular risk.

Higher serum enterolactone concentrations have also been associated prospectively with lower risks of acute coronary events and cardiovascular mortality [87,88]. Human intervention studies further show associations between urolithin, anthocyanin-derived, or flavan-3-ol-derived metabolites and vascular or lipid-related responses [76,77,83]. These findings support metabolite-centered hypotheses but remain mainly correlational.

Overall, evidence is strongest for intermediate outcomes such as endothelial function, blood pressure, plasma lipids, inflammatory markers, and mitochondrial biomarkers. Direct evidence that individual metabolites reduce myocardial infarction, stroke, heart failure, or cardiovascular mortality remains limited. Circulating metabolites should therefore be regarded as biomarkers of exposure and candidate mediators until causal effects are demonstrated at physiologically relevant concentrations. Methodological priorities for resolving these uncertainties are considered in Section 7 and Section 8.

## 5. Whole Foods Versus Isolated Polyphenol Supplementation—A Critical Perspective

Polyphenol-rich dietary patterns have been associated with lower cardiovascular risk, but it remains unclear how much of this association can be attributed to individual polyphenols. Whole foods and isolated supplements represent fundamentally different forms of exposure. They differ not only in polyphenol dose and composition but also in food matrix, accompanying nutrients, pattern of consumption, gastrointestinal release, microbial transformation, and duration of exposure.

Fruits, vegetables, tea, cocoa products, nuts, and extra-virgin olive oil contain mixtures of polyphenols together with fiber, unsaturated lipids, vitamins, minerals, and other bioactive compounds. These components can modify polyphenol release, stability, absorption, microbial metabolism, and elimination. Fibers may prolong intestinal exposure and provide substrates for microbial fermentation, while dietary lipids can alter the gastrointestinal processing of selected relatively lipophilic compounds. Other food constituents may stabilize polyphenols or modify the intestinal environment in which they are metabolized. The resulting food matrix effect is therefore biologically plausible, but the contribution of each component is difficult to isolate in human studies. As discussed in Section 3, whole foods and supplements may also generate different profiles of host- and microbiota-derived metabolites.

Trials of purified compounds and concentrated extracts have produced variable results. Resveratrol is a useful example. Despite extensive experimental evidence, clinical studies have reported heterogeneous effects on blood pressure, lipid variables, glucose metabolism, endothelial function, and inflammatory biomarkers. Differences in dose, formulation, participant characteristics, intervention duration, and rapid presystemic metabolism probably contribute to this variability. Current clinical evidence does not support a single standardized resveratrol regimen for cardiovascular prevention [80].

Green tea interventions provide a related but distinct example. Meta-analyses of randomized trials indicate modest reductions in total and low-density lipoprotein cholesterol, but the magnitude of the response varies among studies [81]. These results apply to specific tea preparations and extracts and should not be generalized to all catechin-containing products. Likewise, effects observed with brewed green tea should not be treated as equivalent to those of concentrated epigallocatechin gallate supplements.

Concentrated supplementation may also introduce safety concerns that are uncommon at normal dietary intake. A systematic assessment of randomized trials identified liver enzyme elevations in a small number of participants receiving green tea extracts, although serious liver-related adverse events were not reported and the estimated risk remained uncertain. Later case evidence has continued to associate concentrated green tea extracts, particularly high-dose products, with uncommon but potentially important liver injury. The safety profile of brewed tea should therefore not be assumed to apply directly to concentrated extracts or products used under fasting conditions [89].

Comparative evidence does not support a simple distinction in which whole foods are effective and purified extracts are ineffective. In a meta-analysis of 46 randomized trials involving 2494 adults with cardiometabolic risk, whole polyphenol-rich foods were associated with reductions in systolic and diastolic blood pressure, whereas purified extracts showed effects on total cholesterol and triglycerides. Neither intervention category consistently improved every outcome, and the findings were limited by substantial heterogeneity and risk of bias [82].

Whole foods may nevertheless offer important practical advantages. They provide repeated exposure to multiple bioactive compounds within dietary patterns that can replace foods rich in sodium, refined carbohydrates, saturated fat, or energy. Supplements add a compound to the existing diet without necessarily improving overall dietary quality. Conversely, standardized extracts permit more accurate dosing and can help identify compound-specific effects. Whole-food and supplement studies should therefore be viewed as complementary rather than interchangeable.

Overall, cardiovascular benefits are more consistently supported for polyphenol-rich foods consumed as part of established healthy dietary patterns than for routine use of isolated high-dose supplements. This conclusion does not prove that food matrix synergy is solely responsible for the difference. Whole-food studies also capture nutrient substitution, repeated exposure, behavioral changes, and interactions with the background diet. The evidence therefore supports a systems-based interpretation in which polyphenols act within a network of dietary, microbial, metabolic, and behavioral influences rather than through a universal single-compound, single-target mechanism.

## 6. Clinical Evidence and the Translational Gap

Despite extensive experimental and epidemiological evidence, the translation of polyphenol research into consistent clinical cardiovascular benefits remains incomplete. Interpretation requires a clear distinction among dietary-pattern trials, interventions with polyphenol-rich foods, trials of standardized extracts or purified compounds, and observational studies. These designs address different questions and should not be treated as equivalent. Quantitative findings are reported for the major trials and meta-analyses most relevant to the translational scope of this review, whereas broader compound-specific summaries are available elsewhere [6,74,80,81,82].

### 6.1. Evidence from Dietary-Pattern Trials

The strongest clinical evidence concerns complete dietary patterns rather than isolated polyphenols. In the reanalyzed PREDIMED trial, 7447 participants at high cardiovascular risk were assigned to a Mediterranean diet supplemented with extra-virgin olive oil, a Mediterranean diet supplemented with nuts, or advice to follow a lower-fat diet. Over a median follow-up of 4.8 years, the adjusted hazard ratios for the primary composite outcome were 0.69 for the extra-virgin olive oil group and 0.72 for the nut group compared with the control group [90].

PREDIMED provides strong evidence for a Mediterranean dietary pattern supplemented with extra-virgin olive oil or nuts, but it does not isolate the effects of polyphenols. The interventions simultaneously modified intake of unsaturated fatty acids, fiber, micronutrients, phytosterols, and numerous other food-derived compounds. In addition, the original trial contained deviations from the intended randomization process, although sensitivity analyses in the reanalysis produced similar findings [83]. The findings therefore support polyphenol-rich dietary patterns, not the conclusion that olive oil or nut polyphenols alone reduced cardiovascular events [90].

### 6.2. Evidence from Polyphenol-Rich Foods and Extracts

Randomized interventions with cocoa, tea, berries, apples, grapes, and olive oil more commonly assess intermediate outcomes than clinical cardiovascular events. Reported effects include improvements in flow-mediated dilation, modest reductions in blood pressure, changes in plasma lipids, reduced susceptibility of low-density lipoproteins to oxidation, and improved measures of high-density lipoprotein function. These outcomes can support biological plausibility but cannot by themselves demonstrate prevention of myocardial infarction, stroke, heart failure, or cardiovascular death.

Flavan-3-ol-rich interventions provide comparatively consistent evidence for improved endothelial function. As discussed in Section 4.1, both acute and repeated interventions have improved flow-mediated dilation, while effects on blood pressure tend to be larger in participants with elevated baseline values. However, the considerable variation among foods, doses, comparators, and participant populations limits the identification of a single effective exposure.

Controlled olive oil studies also support effects on lipoprotein-related outcomes. In a randomized crossover trial, polyphenol-rich olive oil improved high-density lipoprotein cholesterol efflux capacity compared with polyphenol-poor olive oil, while also altering high-density lipoprotein composition and increasing its content of olive oil-derived metabolites [91]. This finding supports a role for the polyphenol fraction within olive oil, but the intervention was short and assessed a functional biomarker rather than cardiovascular events.

### 6.3. Evidence from Isolated Compounds

Trials of isolated compounds generally report inconsistent or modest effects on surrogate outcomes. Variation in formulation, dose, treatment duration, baseline risk, medication use, and metabolic phenotype complicates comparisons among studies. In addition, purified compounds can generate exposure profiles that differ substantially from those produced by the corresponding food.

The COSMOS trial provides the most informative test of long-term cocoa extract supplementation. A total of 21,442 older adults received cocoa extract containing 500 mg of flavanols per day, including 80 mg of epicatechin, or placebo for a median of 3.6 years. Cocoa extract did not significantly reduce the prespecified composite of total cardiovascular events in the intention-to-treat analysis. Cardiovascular mortality was lower as a secondary outcome, while per-protocol analyses suggested a possible reduction in total cardiovascular events. These secondary and adherence-based findings are hypothesis-generating and do not change the neutral result for the primary outcome [92].

COSMOS therefore illustrates both the potential and limitation of supplementation trials. Favorable effects on vascular biomarkers observed in smaller, shorter studies do not necessarily translate into fewer clinical events. The findings also cannot be directly compared with chocolate or cocoa foods because the intervention was a standardized cocoa extract rather than a conventional food product.

### 6.4. Observational Evidence

Prospective cohort studies generally report inverse associations between greater consumption of flavonoid- or polyphenol-rich foods and cardiovascular risk. However, higher polyphenol intake is often associated with greater consumption of fruits, vegetables, tea, whole grains, legumes, nuts, and olive oil, as well as other health-related behaviors. Residual confounding therefore remains difficult to exclude. These associations may also be influenced by healthy-user bias and reverse causality, because individuals who consume more polyphenol-rich foods may follow healthier lifestyles overall, while existing health conditions may alter dietary behavior.

Measurement error presents an additional challenge. Food-frequency questionnaires may not capture variation in cultivar, ripeness, processing, storage, cooking, or food formulation, all of which can influence polyphenol content. Polyphenol databases also differ in compound coverage, analytical methods, and food-consumption data. Biomarker-based assessment can improve estimation of exposure but introduces other sources of variation, including recent intake, renal function, host metabolism, and microbial conversion.

Observational associations should consequently be regarded as complementary to randomized evidence rather than as proof that individual polyphenols prevent cardiovascular disease. Their main value lies in identifying dietary patterns, food sources, dose ranges, and populations suitable for controlled testing.

### 6.5. Integrated Interpretation

The clinical evidence does not support a universal cardioprotective effect for all polyphenols. Instead, the clearest benefits occur for selected polyphenol-rich foods and dietary patterns, particularly when consumed repeatedly and when they replace less favorable foods. Isolated compounds and extracts may improve selected risk markers, but convincing evidence for reductions in major cardiovascular events remains limited.

The translational gap reflects more than low bioavailability. It also arises from differences between experimental and human exposure, incomplete identification of active metabolites, short intervention periods, weak comparators, heterogeneous populations, limited adherence, and reliance on surrogate endpoints. Cardiovascular effects may further depend on baseline risk. A modest reduction in blood pressure may be more detectable and clinically relevant in participants with hypertension than in healthy normotensive volunteers.

The most defensible interpretation is therefore neither that polyphenols are ineffective nor that they act as broadly protective pharmacological agents. Polyphenols should instead be considered one group of potentially active components within foods and dietary patterns whose effects depend on chemical form, dose, matrix, background diet, metabolic capacity, and clinical context.

## 7. Limitations and Methodological Challenges in Human Studies

Several methodological limitations restrict comparisons among human polyphenol studies and weaken conclusions about cardiovascular efficacy.

### 7.1. Intervention Heterogeneity

The term polyphenol intervention includes whole foods, beverages, enriched foods, complex botanical extracts, purified compounds, and directly administered microbial metabolites. These interventions differ in chemical composition, dose, food matrix, energy content, co-nutrients, contaminants, and bioavailability. Grouping them into a single category can produce pooled estimates with limited biological meaning.

Incomplete chemical characterization is common. Reporting only total polyphenol content does not identify individual compounds, stereoisomers, degradation products, or changes during storage and digestion. Different analytical assays can also produce non-equivalent estimates of total polyphenols. Future trials should provide detailed compositional data and, where possible, stability and batch-to-batch measurements.

### 7.2. Comparator Selection and Blinding

Suitable placebos are difficult to design for foods and beverages. Removing polyphenols can alter color, taste, texture, caffeine content, energy, or other bioactive constituents. Conversely, comparing one polyphenol-rich food with no intervention may capture food replacement or behavioral effects rather than a specific polyphenol effect.

Blinding is often incomplete because participants can identify the intervention from sensory characteristics. Adherence may also differ between familiar foods and capsules. Trials should report the success of masking where possible and use objective exposure biomarkers in addition to self-reported adherence.

### 7.3. Small Samples, Short Duration, and Surrogate Outcomes

Many trials include relatively few participants, last from several hours to a few weeks, and assess multiple outcomes without a clearly prioritized primary endpoint. Such designs can evaluate acute vascular or metabolic responses but cannot establish long-term cardiovascular prevention.

Flow-mediated dilation, blood pressure, plasma lipids, inflammatory markers, and oxidative biomarkers are informative intermediate outcomes, but their clinical meaning differs. A statistically significant change in a biomarker should not automatically be described as cardioprotection. Studies should report effect sizes and confidence intervals and consider whether the observed change is large enough to be physiologically or clinically relevant.

### 7.4. Exposure Misclassification and Background Diet

Nominal intervention dose does not necessarily represent the amount that is bioaccessible, absorbed, or delivered to target tissues. Differences in food matrix, gastrointestinal processing, microbiota, and host metabolism can produce distinct internal exposures from the same administered dose.

The background diet is equally important. Participants may already consume substantial amounts of the tested polyphenol class, reducing the contrast between intervention and control groups. Dietary substitution can also confound interpretation. Adding berries to the diet may have a different effect from using berries to replace a refined dessert, even when the nominal polyphenol dose is the same.

Repeated dietary records, validated intake instruments, washout procedures where appropriate, and circulating or urinary biomarkers should be used to characterize both intervention exposure and background intake.

### 7.5. Interindividual Variability

As discussed in Section 3.5, participants differ in intestinal absorption, Phase II metabolism, renal elimination, and microbial conversion. Consequently, a uniform oral dose may produce widely different circulating metabolite profiles. An average treatment effect can therefore conceal responses limited to participants who achieve sufficient exposure to a particular metabolite.

Most studies remain too small to distinguish true effect modification from chance subgroup findings. Post hoc classification of participants as responders and nonresponders is particularly vulnerable to regression to the mean and multiple testing. Metabolic subgroup analyses should be prespecified, use validated definitions, and include adequate numbers within each subgroup.

### 7.6. Multiplicity and Selective Interpretation

Polyphenol trials frequently measure many vascular, metabolic, inflammatory, and oxidative outcomes at several time points. Without prespecified primary outcomes and appropriate control of multiplicity, isolated statistically significant findings may occur by chance.

Selective emphasis on favorable secondary outcomes is especially problematic when the primary outcome is neutral. The COSMOS findings illustrate the need to preserve this hierarchy: the primary composite outcome was not significantly reduced, while the cardiovascular mortality result was secondary and should be interpreted accordingly [92].

### 7.7. Formulation and Safety

Formulation can affect both efficacy and safety. Concentrated extracts may produce higher peak exposures than foods, while fasting administration can differ from intake with a meal. Products may also vary in purity, excipients, and chemical stability. Safety reporting is often less detailed than efficacy reporting. Trials should systematically assess adverse events, liver and renal markers where relevant, medication interactions, and reasons for withdrawal. Findings from high-dose extracts should not be extrapolated to normal food consumption, and the established safety of a food should not be assumed for a concentrated supplement.

## 8. Future Perspectives: Toward Precision Nutrition and Mechanism-Guided Intervention

Future research should move beyond one-size-fits-all supplementation toward integrated, mechanism-guided, and stratified approaches. Precision nutrition frameworks should combine measures of internal exposure with relevant features of the gut microbiome, host metabolism, and genetic variation to identify factors associated with individual responses. However, although interindividual variability in polyphenol metabolism is well established, the clinical value of microbial, genetic, or metabolomic markers for predicting cardiovascular responses has not yet been validated.

Randomized controlled trials should include adequate sample sizes, longer follow-up, and clinically relevant primary endpoints rather than relying mainly on surrogate markers. Detailed and standardized characterization of polyphenol composition in interventions is also needed to improve reproducibility and comparisons across studies. Targeted metabolomics should be used to confirm exposure to parent compounds and metabolites, while broader approaches, including metagenomics and lipidomics, may help explain variability when guided by specific hypotheses.

Greater emphasis should be placed on food-based interventions and dietary patterns that reflect real-world exposure. Purified compounds and standardized extracts remain valuable for defining mechanisms and dose–response relationships, but their findings should not be directly extrapolated to whole foods. Ultimately, studies should determine which polyphenol-rich foods or preparations are beneficial, for whom, and through which measurable metabolic pathways.

## 9. Conclusions

Dietary polyphenols represent one of the most intriguing bridges between nutrition and cardiovascular health. Their chemical diversity translates into a wide spectrum of biological actions, ranging from modulation of endothelial function and vascular tone, through regulation of mitochondrial homeostasis and redox signaling, to attenuation of inflammation, platelet activation, and lipid metabolism. These findings support the biological plausibility of cardiovascular effects but do not establish that dietary polyphenols independently reduce cardiovascular risk.

Yet, the path from molecular insight to clinical application remains complex. Human evidence is most consistent for polyphenol-rich foods consumed within healthy dietary patterns, although these effects cannot be attributed specifically to polyphenols. In contrast, trials with isolated compounds or supplements often yield modest and inconsistent results, underscoring the importance of food matrix effects, dose relevance, and host–microbiota interactions. The translational gap is therefore not a failure of polyphenols per se, but a reflection of their chemical heterogeneity, variable bioavailability, and the profound influence of individual metabolic capacity.

Cardiovascular responses should be understood as the integrated outcome of parent molecules, host-derived conjugates, microbial metabolites, and co-occurring food constituents. This complexity calls for a paradigm shift: polyphenols should no longer be treated as a single functional class, nor evaluated solely through the activity of their ingested forms. Instead, future research must embrace metabolite profiling, microbial phenotyping, and physiologically relevant mechanistic models to unravel which foods, preparations, and combinations are most effective, and in which populations.

Ultimately, dietary polyphenols exemplify the promise of precision nutrition. By integrating chemistry, microbiology, and clinical science, they can be positioned not as isolated “magic bullets,” but as part of holistic dietary strategies that support cardiovascular resilience. With carefully designed interventions and personalized approaches, polyphenols may contribute to cardiovascular health, particularly as part of balanced, polyphenol-rich dietary patterns. Thus, dietary polyphenols should be considered not as isolated pharmacological agents, but as components of broader dietary strategies, while their effects on major cardiovascular outcomes require further confirmation in well-designed clinical studies.

## Figures and Tables

**Table 1:** Comparison of bioavailability and metabolism, mechanisms of action, and human/clinical evidence addressed in previous reviews and the present review.

Main Focus	Bioavailability & Metabolism	Mechanism of Action	Human/Clinical Evidence	Key Limitation	Ref.
Polyphenols and CVD prevention	Discussed	Partial discussion	Discussed	Active compounds responsible for the observed effects uncleared; Limited understanding of bioavailability and ADMET properties of polyphenols	[13]
Dietary polyphenol intake and cardiovascular protection	Detailed discussion	Discussed	Discussed	Mainly epidemiological evidence; limited clinical intervention data and limited integration of bioavailability/metabolite-based mechanisms	[14]
Mechanisms beyond antioxidant activity	Partial discussion	Detailed discussion	Partial discussion	Limited clinical translation	[15]
Endothelial dysfunction and CVD	Partial discussion	Detailed discussion	Partial discussion	Focus on endothelial cells	[16]
Atheroprotective effects of polyphenols	Partial discussion	Detailed discussion	Discussed	Focused on atherosclerosis	[10]
In vitro cardioprotective effects	Not addressed	Detailed discussion	Not addressed	Lack of clinical evidence	[7]
Polyphenols in cardiovascular prevention and treatment	Partial discussion	Detailed discussion	Discussed	Focus on selected compounds	[17]
Polyphenol metabolism and cardioprotection	Detailed discussion	Detailed discussion	Limited discussion	Limited integration with clinical outcomes	[6]
Comprehensive review	Detailed discussion	Detailed discussion	Detailed discussion	Dietary sources, bioavailability, gut-microbiota metabolism, molecular mechanisms, and clinical evidence; translational gap highlighted	Current review

## Data Availability

No new data were created or analyzed in this review. Data sharing is not applicable to this article.
